# Tendinopathies in Video Gaming and Esports

**DOI:** 10.3389/fspor.2021.689371

**Published:** 2021-05-28

**Authors:** Caitlin McGee, Kevin Ho

**Affiliations:** ^1^Health Providence, Los Angeles, CA, United States; ^2^School of Health Sciences, The University of Sydney, Darlington, NSW, Australia

**Keywords:** esports, tendinopathy, tendon rehabilitation, musculoskeletal injury, esports athletes

## Introduction

Esports, defined by Wagner as “an area of sport activities in which people develop and train mental or physical abilities in the use of information and communication technologies,” is a new and intriguing arena of competition (Wagner, 2006). While the esports industry has grown significantly in the past decade, garnering over 450 million viewers worldwide and upwards of $1 billion in revenue in 2019 alone, the healthcare infrastructure to support this industry has grown at a much slower rate (Newzoo, 2019). Unlike their traditional sports counterparts, not all professional esports competitors have access to team physicians, physical therapists, or athletic trainers. Instead, they seek care from independent medical professionals who may be less familiar with the physiological demands of esports (Khan, 2017; Joe, 2021; Samples, 2021). While not an exhaustive accounting, these demands may include unique ergonomic and postural considerations across different game types, wrist and hand muscular endurance, and potential physiological effects related to psychological stress during competition. In the absence of a substantial body of research on health, injuries, and performance in esports, medical professionals rely on basic biomechanical principles and research from related fields.

Esports competitors have practice and competition obligations tied to their compensation. They use a variety of input devices, such as mice, keyboards, and console controllers, which vary in sensitivity or force required for interaction, types of movements performed, and positions of most-used buttons or keys. Differences in the size and weight of the mouse, force attenuation of keyboard keys, or sensitivity of controller joysticks may result in variations in physical load from player to player. Professional players encounter competitive strains including travel, sleep disruption, and performance-related stress as traditional sports athletes do (Bonnar et al., 2019; Poulus et al., 2020). As the demands of esports involve novel stressors in addition to these established competitive strains, analogous research is insufficient to provide adequate clinical practice guidelines in the long term. Establishing such guidelines requires esports-specific research identifying risk factors for injury, establishing injury prevalence data, validating diagnostic tools, and evaluating programming for injury prevention and intervention.

**Figure 1:**
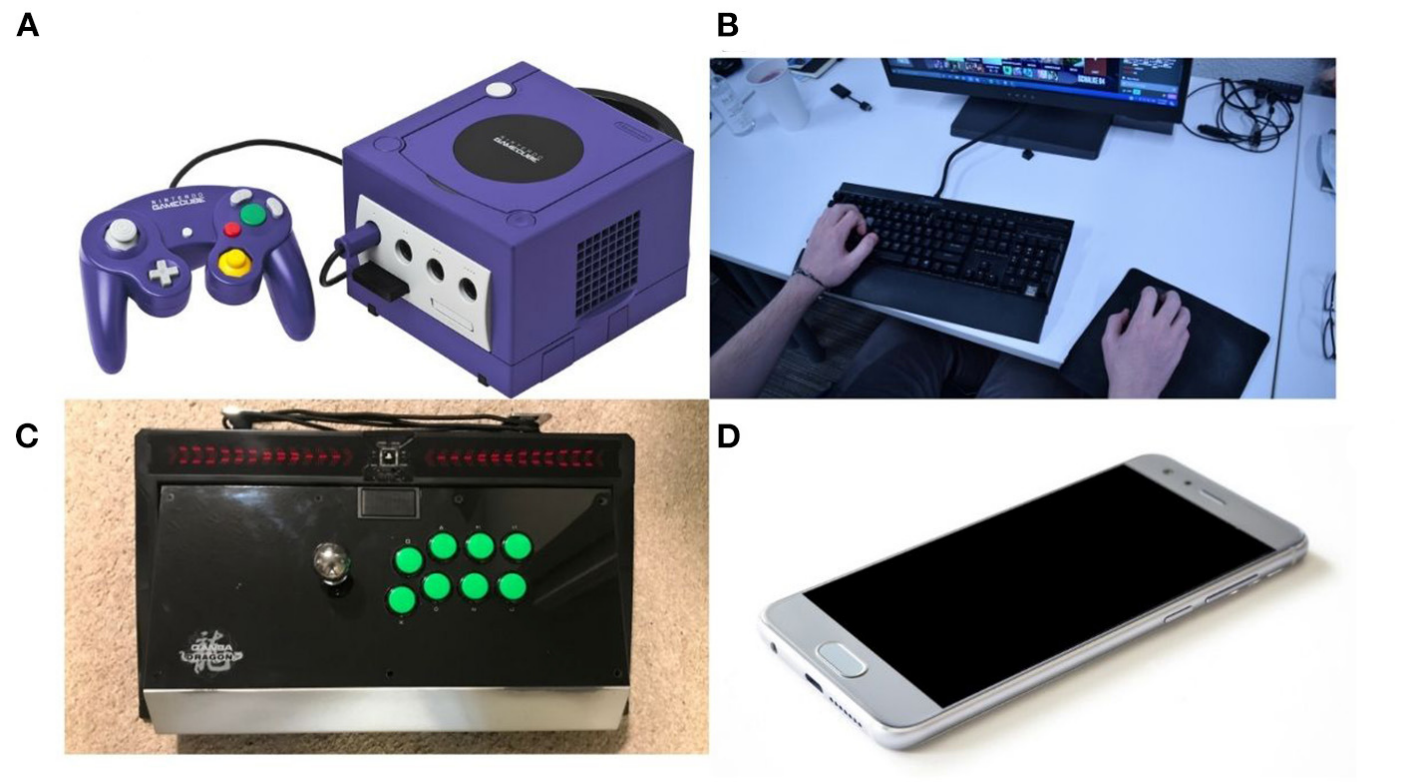
Examples of controllers used in esports. **(A)** Gamecube controller, a typical example of a console controller. **(B)** Mouse and keyboard, used for personal computer (PC) gaming. **(C)** Arcade-style stick, most often used for console gaming. **(D)** Smartphone, used for mobile gaming.

Within the already-specific field of research on esports, there must be an even greater degree of specificity to address the generalizability of injury research or lack thereof, given the diversity of game types, input devices, and playstyles. At present, no data exists differentiating injury rates among players who use console controllers, mice and keyboards, arcade-style sticks controllers, and mobile devices such as smartphones or tablets. Examples of these devices are shown in Figure 1.

Using the basic principles of biomechanics, medical professionals may reasonably assume general differences in which muscle groups and joints are likely to experience the most strain with each of those devices. However, in the absence of esports-specific and even game- and device-specific research, no concrete determination of best medical practices can be established.

## Injury Risk Factors in Esports

While such research is developed, existing research on injuries, ergonomics, and posture in office workers is well-established and may provide a basis for preliminary best practices, given that many, although not all, esports competitors use similar input devices (e.g., mouse, keyboard, monitor) as office workers. Per DiFrancisco-Donoghue et al. (2019) esports competitors exhibit many of the same pain and injury patterns as office workers. In both populations, these injury patterns include neuropathic and tendinopathic conditions, including epicondylopathies (wrist flexor and extensor tendinopathies), de Quervain's tenosynovitis, and intersection syndrome.

A number of occupational factors have been identified as contributing to increased risk for the development of upper limb tendinopathies, including repeated or sustained wrist bending, repeated twisting or pushing motions, non-neutral wrist postures during work activity, and repetitive forceful motions (Shiri et al., 2006; Petit Le Manach et al., 2011; Shiri and Viikari-Juntura, 2011; Herquelot et al., 2013). While office workers routinely perform 130–180 keyboard and mouse inputs, or actions per minute (APMs), over the course of an 8 h workday (Szeto et al., 2005), esports competitors perform up to 500–600 APMs and regularly train for 5–10 hours per day (Lewis et al., 2011; DiFrancisco-Donoghue et al., 2019). This results in repetitive loading to a degree that may result in elevated risk of tendinopathic conditions. Sustained loading can also contribute to elevated risk. A variety of ergonomic considerations which may affect repetitive and sustained loads exist for esports competitors is provided in Table 1.

**Table 1:** Ergonomic considerations for repetitive and sustained loading and potential elevated injury risk.

**Ergonomic consideration**	**Associated physiological consideration**
Keyboard key force attenuation	Repetitive movement against resistance
Keyboard and mouse angle	Sustained wrist ulnar/radial deviation
Mouse size	Sustained wrist extension angle
Mouse grip type	Sustained forearm/wrist/hand muscle contraction, sustained wrist position in multiple planes
Lack of arm support	Sustained postural, shoulder, forearm, and wrist muscle contraction for stability
Most commonly-used keys	Sustained wrist position in multiple planes, repetitive finger movement (varying per key use)
Infrequent whole-body movement	Sustained postural muscle contraction, sustained loading of passive structures (joints, ligaments)

Lifestyle factors, including smoking and obesity, also contribute to elevated risk for upper limb tendinopathy (Shiri et al., 2006; Herquelot et al., 2013). Early research suggests that esports competitors have higher body-fat percentage and have less lean body mass than age- and gender-matched peers (DiFrancisco-Donoghue et al., 2019). This is likely due to lower activity levels, although early data suggests top-ranked esports competitors are more physically active compared to lower-ranked players (Bayrakdar et al., 2020; Trotter et al., 2020).

## Current Tendon Research and Potential Applicability

Tendinopathies are thought to have a pathological continuum involving three phases. These in order from least to most severe are: reactive tendinopathy, tendon disrepair, and degenerative tendinopathy. As a continuum, a tendon may improve or regress through these stages based on the load placed on it. Tendons further down this continuum, toward the degenerative stage due to continual overloading, have less potential to return back to a normal healthy tendon.

Most tendinopathies can return to normal if managed early on with optimal loading, where the load is adequate for proper tendon healing and restructuring (Rio et al., 2015a). It is a balance between complete rest where muscles and tendons actually get weaker, and overload where the tendon's condition worsens. Importantly, this model also highlights why interventions that purely target pain (e.g., steroid injections) and immobilization (i.e., splinting) have been ineffective (Cavaleri et al., 2016; Ippolito et al., 2020).

Optimal loading of a tendon involves the combination of graded activity, and strengthening and possibly motor control. Rehabilitation programs that have been successful in terms of pain reduction and return to sport outcomes usually include strength training. For strength outcomes, neither concentric (shortening) nor eccentric (lengthening) nor isometric (static) contractions have been found to be superior (Couppé et al., 2015; Quinlan et al., 2019). However, isometric contractions have been found to also provide immediate pain relief (Rio et al., 2015b). Recent research has also found that the coordination and timing of muscle contractions are often impaired in tendinopathies, and propose that correcting these impairments *via* tendon neuroplastic training may bring further improvements in pain (Rio et al., 2015a). Strategies to improve motor control during strength training include external pacing with a metronome or visual stimulus.

Epicondylopathies are distinct type of tendinopathy which occur at the elbow. Most of the forearm muscles which control the fingers and wrist attach proximally via a common tendon to the elbow. Lateral epicondylalgia refers to a tendinopathy of the tendon complex for the finger and wrist extensors. Medial epicondylalgia affects the similar tendon complex for the finger and wrist flexors. Therefore, management of these commons typically target the finger and wrist movements that these muscles are responsible for, rather than the elbow movement. Based on the high APMs cited earlier, tendinopathies of the distal tendons of these muscles which connect to the fingers and wrist may be more common in esports competitors than proximal tendinopathies, but additional research is required to establish this with any degree of certainty.

## Conclusion and Future Research

In the absence of more concrete research, medical professionals in esports must rely on the data available to them in the form of experience, expert opinion, and relevant research in other populations. As established by research on office workers, esports competitors are subject to the kinds of repetitive loads which increase risk for tendinopathy. Extensive research exists on all facets of tendon pathologies, from prevention to development to treatment.

Given the current dearth of esports-specific research, medical professionals working in the field should apply existing research in related fields to provide care for esports competitors at present. Significant research is needed into injury prevalence, validation of injury-prediction measures, and effectiveness of interventional and preventional programming. Research is also needed to assess the effects of mouse size and weight, controller size and weight, key force attenuation, common movement patterns across input devices, and other esports-specific ergonomic concerns. Additionally, research on the effects of supplements, sleep, travel, and exercise on esport competitor performance, not just health, is necessary to establish clinical practice guidelines for medical professionals and best practices for coaches and teams.

In considering specific research on tendinopathy in esports competitors, we propose several areas of potential immediate interest. With regards to diagnosis, MRI and ultrasound techniques may be used to identify pathophysiological changes (Warden et al., 2007) in tendinopathies acquired from high-impact activities such as jumping and running. However, these changes do not necessarily correlate with symptoms or diagnosis of tendinopathy based on clinical examination (Khan et al., 1997; Giza et al., 2013). Given the difference in potential mechanism of injury in esports competitors, namely low-impact but highly repetitive activities, the association between imaging-identified pathophysiological changes and clinically diagnosed tendinopathies may differ and should be investigated. With regards to prevention, the development and validation of predictive tests for tendinopathy risk incorporating ergonomics, strength, endurance, and lifestyle habits would provide the opportunity for preventative programming or earlier intervention. Finally, while isometric, concentric, and eccentric contractions may all be part of an appropriate treatment plan in tendinopathies developed from high-impact sports, their effectiveness in treatment of low-impact repetitive strain injuries such as those esports competitors may experience should also be established.

## Author Contributions

CM conceived of the presented idea. CM and KH developed the outline, conducted appropriate research and reviews in conjunction with each other, and completed the manuscript together. All authors contributed to the article and approved the submitted version.

## Conflict of Interest

The authors declare that the research was conducted in the absence of any commercial or financial relationships that could be construed as a potential conflict of interest.
